# “Sometimes It Felt Great, and Sometimes It Just Went Pear-Shaped”: Experiences and Perceptions of School Nurses’ Motivational Interviewing Competence: A Convergent Mixed-Methods Study

**DOI:** 10.3390/clinpract12030039

**Published:** 2022-05-20

**Authors:** Marianna Moberg, Helena Lindqvist, Susanne Andermo, Åsa Norman

**Affiliations:** 1 Department of Global Public Health, Karolinska Institutet, 171 77 Stockholm, Sweden; susanne.andermo@ki.se (S.A.); asa.norman@ki.se (Å.N.); 2 Department of Clinical Neurosciences, Karolinska Institutet, 171 77 Stockholm, Sweden; helena.lindqvist@ki.se; 3 Department of Neurobiology, Care Science and Society, Karolinska Institutet, 141 52 Huddinge, Sweden; 4 Department of Physical Activity and Health, The Swedish School of Sport and Health Sciences, 114 33 Stockholm, Sweden; 5 Department of Psychology, Stockholm University, 106 91 Stockholm, Sweden

**Keywords:** motivational interviewing, MITI-4, self-rated competence, school nursing, client perspectives

## Abstract

In this convergent mixed-methods study, the aim was to explore how objective and subjective quality ratings of school nurses’ motivational interviewing (MI) correlate whilst also considering the perceptions of delivering and participating in the same MI sessions. Quantitative and qualitative data were derived from seven intervention schools participating in the Healthy School Start Plus parenting support intervention. School nurses were trained in MI and conducted an MI session with parents of 6–7-year-old children to discuss children’s physical health and development. Quantitative data comprised objective ratings of school nurses’ MI competence using the Motivational Interviewing Treatment Integrity 4.2 [MITI-4] protocol, as well as parents’ and school nurses’ subjective ratings of the MI sessions. Qualitative data comprised semi-structured interviews with parents and school nurses about their perceptions of the MI sessions. First, quantitative data were analysed using Spearman’s rank correlation, and qualitative data were analysed using content analysis. Next, quantitative and qualitative findings were merged. Our findings suggest that school nurses’ MI performances were rated and perceived as valuable and family-centred by both school nurses and parents who had left the meeting feeling motivated and empowered to promote their children’s healthy behaviours. Nonetheless, school nurses were critical to their own MI technical performance, and they found that reflections were easier to deliver and to self-rate. Overall, MITI ratings were the lowest and parents’ ratings were the highest. Future studies should focus on relating clients’ subjective ratings of MI with clients’ behavioural outcomes.

## 1. Introduction

Childhood overweight and obesity is among the major threats to public health globally, increasing the risk of non-communicable diseases [1]. Persistent energy imbalances are modifiable factors contributing to abnormal growth development. Eating and physical activity behaviours established during childhood are likely to track individuals through adolescence and into adult life [2,3,4]. Close family and the surrounding environment are crucial influencers when it comes to younger children’s energy-balance-related behaviours [5]. Primary school in Sweden is compulsory. This provides school nurses with an exceptional opportunity to reach parents of all school-aged children with health-promoting activities [6]. Promoting healthy food and physical activity behaviours and thus preventing harmful weight development over time, is part of school nurses’ core mission [7,8]. Person-centred care has been described as care provided with a holistic focus and with an emphasis on inter-relational aspects, such as empathy, respect, engagement, relationship, and communication [9]. Both the international code of ethics for nurses [10] and national guidelines for school nurses [8,11] emphasize the importance of a person-centred approach when providing health care. Motivational interviewing (MI) is a person-centred counselling method [12] and has been shown to be an effective method to support behavioural change in various health contexts [12]. In general, there are four important overarching MI skills practitioners need master to reach proficiency. First, the technical construct of MI comprises practitioners’ ability to cultivate clients’ change talk and to soften clients’ talk about sustained behaviours. Second, the relational construct of MI includes clinicians’ competence in showing empathy and encouraging partnership with clients [13]. The technical constructs in MI are characterised by practitioners’ ability to reflectively listen with the deliberate purpose in order to evoke or soften clients’ talk in the direction of the set behavioural goals [13]. The relational construct of *partnership*, on the other hand, reflects practitioners’ ability to engage clients in the conversation [13], whereas the construct of *empathy* in MI is characterised by practitioners’ ability to perceive and communicate understanding of other people’s feelings and their attached meaning [14]. High ratings of clinicians’ *empathy* have been associated with positive behaviour change in several studies [15], and clinicians’ reflective listening is one of the most important skills with regard to empathy in MI [13].

There is a well-established curriculum of how to teach the techniques and spirit of MI [16]. Nonetheless, studies have shown that MI can be a difficult method for clinicians to learn and uphold over time [12,17,18]. Assessment of clinicians’ competence and fidelity to delivering a method is a crucial part of understanding whether and how a method works and thus an important part of the development of all evidence-based practices [19]. Treatment fidelity refers to the extent to which an intervention was implemented as intended with regard to clinicians’ adherence and competence [20]. There are multiple tools for evaluating MI competence, and the Motivational Interviewing Treatment Integrity (MITI) protocol is the most used and evaluated [21,22]. Criteria for MI providers’ competence in *cultivating change talk* and *empathy* are described on a five-step competence ladder in the MITI protocol. For high scores of *cultivating change talk*, clinicians should repeatedly give attention to client’s talk about willingness and reasons for changing the targeted behaviour. Regarding *empathy*, high scores are achieved when clinicians make accurate and reflective assumptions regarding clients’ perspective and worldview [23]. A commonly used method to assure MI quality in clinical practice is for MI practitioners to evaluate their own performance [20]. Nonetheless, previous research indicates that MI practitioners’ self-assessments do not correlate with objective assessments [24,25]. One perspective that is overlooked is clients’ perceptions and experiences of participating in MI sessions [26]. Two qualitative studies have concluded that clients perceive clinicians’ *empathy* skills as fundamental in promoting clients’ self-efficacy [27] and to facilitate change [28]. Nonetheless, no published studies have been found comparing clinician, client and objective experiences and perceptions.

### 1.1. Rationale

Objective quality assessment of MI is both reliable and valid [21,22]. Nonetheless, regular MI fidelity assurance can be both time-consuming and costly. One way around this would be for MI practitioners to evaluate their own performance. Nonetheless, previous research indicates that MI practitioner self-assessment can be difficult [24,25]. Furthermore, it is important for understanding the mechanisms of MI to also include provider and client experiences of participating in MI sessions. In addition, the combination of qualitative and quantitative measures with a mixed-methods approach, to shed light on both broader and in-depth aspects of MI performance, are seldom used.

### 1.2. Study Aims

This convergent mixed-methods study explored school nurses’ MI competence using quantitative, objective and subjective ratings assessed by MITI coders, parents, and school nurses, together with qualitative perceptions of delivering and participating in MI sessions as reported by school nurses and parents within the Healthy School Start Plus trial. The study was designed to answer three research questions:

1. How do objective ratings of school nurses’ MI quality correlate with the subjective quality ratings from school nurses and parents?
2. What are school nurses’ and parents’ perceptions of delivering and participating in MI sessions?
3. How do objective and subjective ratings of MI sessions resonate with school nurses’ and parents’ perceptions of the same MI sessions?

## 2. Material and Methods

### 2.1. Design

In this study, we applied a convergent mixed-methods study design (QUAL + QUANT) using both qualitative interview questions and quantitative tools for data collection [29]. Data were obtained from seven intervention schools that were part of the cluster randomised Healthy School Start Plus (HSSP) trial. All methodological details of the trial are described in the published study protocol [30] and briefly below.

### 2.2. Participants

**Figure 1:**
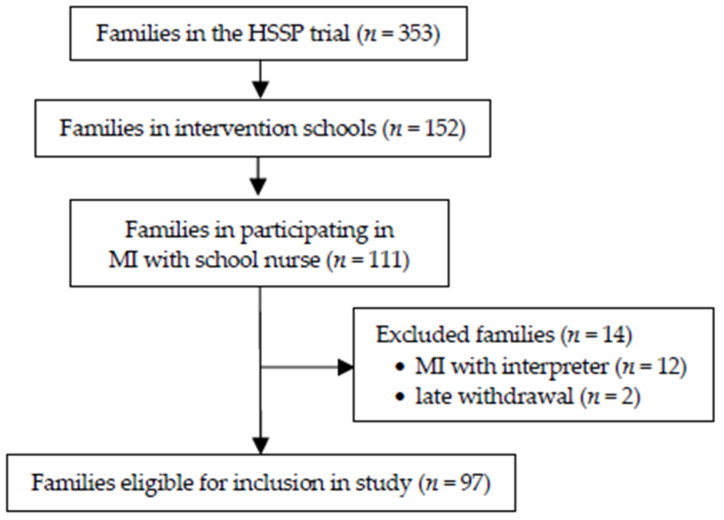
Flow chart of participant recruitment.

The HSSP was a universal six-month intervention (November 2017 to April 2018) targeting parents with children in primary schools in disadvantaged areas in and around Stockholm, Sweden. The aim was to promote healthy dietary and physical activity behaviours and prevent child overweight and obesity through four intervention components: (1) Motivational interviewing: as a complement to the regular health visit during the first primary school year, school nurses delivered health promotion through MI, with parents focusing on dietary and physical activity behaviours. All MI sessions were held at the school nurse’s office at the respective school. (2) Classroom lectures: nine lectures were delivered by teachers to children. (3) Brochure: health information was provided to parents in the form of a brochure. (4) Diabetes risk test: an online type 2 risk diabetes test was administered to parents [31]. Sixteen schools agreed to participate in HSSP and were included in the study; the schools were randomly assigned to either the intervention (*n* = 8) or control (*n =* 8) group. Families of 353 children (aged 5–7 years) agreed to participate in the HSSP trial, including 152 children in the intervention schools and 193 children in control schools (Figure 1). A total of 111 intervention families participated in MI conversations with the school nurse. The present study included 97 families after excluding MI sessions conducted via interpreter (*n =* 12) and late withdrawals (*n =* 2).

#### Motivational Interviewing Training

The MI training for the intervention school nurses consisted of a two-day on-site workshop, practical homework, and two supervisions. The workshops were facilitated by two of the authors (Å.N. and H.L.) and included lectures on MI theory, MI demonstrations, and practical MI exercises, together with lectures held by invited experts focusing on children’s healthy behaviours and parenting practices in relation to food and physical activity. Following the workshop days, all school nurses were assigned homework to conduct and record two MI practice sessions. One of the recordings was transcribed and analysed by the school nurse, focusing on basic MI skills, and then discussed during a group supervision session. The second recording was coded by experts using the MITI coding system, whereby written and oral feedback was given during the individual supervision session. During the intervention, all school nurses had access to all written information provided during the MI training, as well as the possibility to reach out to MINT experts for additional guidance on the method [30].

### 2.3. Ethical Approval

Written consent to participate was obtained from all parents and school nurses. An additional oral consent was obtained from parents before recording the MI conversations and interviews. Ethical approval for the HSSP in accordance with the Declaration of Helsinki [32] as obtained from the Research Ethic Committee in Stockholm, part of the Swedish Central Ethical Review Board (No. 2017/711–31/1).

### 2.4. Data Collection

#### 2.4.1. Quantitative Data

##### MITI Ratings

All conversations were coded by the *Motivational Interviewing Quality Assurance* (MIQA) coding lab at Karolinska Institutet in Sweden using the MITI coding manual version 4.1 [23], objectively assessing the school nurses’ MI performance. Four global ratings are used in MITI: *cultivating change talk*, *softening sustain talk, partnership*, and *empathy*. Each global score is coded using a five-point Likert scale. Furthermore, the MI sessions were coded for MITI-defined behavioural counts, which are ten frequency measures of practitioner behaviours. Counts of *reflections* and *questions* form the basis of the commonly reported *reflection-to-question ratio* score (sum of *reflections* divided by total *questions*) [23]. More details on the coding procedure for this study have been described elsewhere [33]. Three MITI variables were used for the purpose of this study: (1) *cultivating change talk*, (2) *empathy*, and (3) *reflections-to-questions ratio*. Inter-rater reliability between MITI coders was assessed with an intraclass correlation coefficient (ICC) [34]. ICC ranged between 0.66 and 0.85, where ICC scores for *cultivating change talk* and *empathy* were considered good and scores for *reflections* and *questions* were excellent [35].

##### School Nurses’ Ratings

All MI sessions were subjectively rated by the school nurses, who filled in a log after the MI sessions wherein they self-assessed their own MI performance. Two of the variables focused on the global MITI measures: ability to *cultivate change talk* and *empathy*. The two questions were (1) “*In this conversation I demonstrated an effort to encourage the parent to talk about benefits of creating or sustaining healthy food and physical activity behaviours for the child*” and (2) “*In this conversation I demonstrated an effort to understand the parents’ thoughts and feelings*”. These questions were rated on a five-point Likert scale, ranging from 1 = “very little” to 5 = “very much”. The third question answered by the school nurses focused on the perceived *reflections-to-questions ratio* in the MI session: (3) “*In this conversation the proportions of reflections in relation to questions was as follows*”. This question had three possible ratings: 1 = “more questions than reflections”; 2 = “equal number of questions and reflections”; 3 = “more reflections than questions”.

##### Parents’ Ratings

Directly after meeting with the school nurse, parents were asked two questions in a web-based survey regarding the MI session. The questions were (1) *“Did you feel that the school nurse demonstrated an effort to understand your thoughts and feelings?*” and (2) “*Did the school nurse motivate you to create or sustain healthy food and physical activity behaviours for your child?*”. The parents were asked to answer the questions on a five-point Likert scale, ranging from 1 = “very little” to 5 = “very much”. In two cases, both parents attended and rated the same MI session, in which case the average rating of the two parents was used to reflect the parents’ experience.

#### 2.4.2. Qualitative Data

##### Interviews with School Nurses

One of the authors (S.A.) conducted all interviews using a semi-structured interview guide. All participating school nurses (*n =* 7) from the HSSP trial were interviewed face-to-face (*n =* 6) at a location suitable for the interviewee or by specific request from the school nurse via telephone (*n =* 1). All interviews were audio-recorded and transcribed verbatim by a transcription service. Examples of questions that were posed during the interviews were: “*How did you perceive using MI with the parents?*”, “*What was your previous experience using MI?*”, and “*How did you perceive that MI might have influenced parents’ and children’s behaviours?*”.

##### Interviews with Parents

A purposeful sample of parents (*n =* 17) from the intervention schools that had participated in the MI session were interviewed over telephone using a semi-structured interview guide. The sample was selected with a maximum variation strategy [36] regarding parent sex and country of birth, as well as child sex and weight status. A female doctoral student performed the sampling under the supervision of Å.N. by contacting the eligible parents and conducted all interviews. All interviews were audio-recorded and transcribed by a transcription service. Examples of posed questions include: “*How did you experience the conversation with the school nurse?”*, “*How did you experience the school nurse’s approach and attitude?*”, and “*How did the conversation with the school nurse affect your family?”.*

### 2.5. Data Analyses

#### 2.5.1. Statistical Analysis

Spearman correlation coefficient (ρ) with a two-tailed significance level of 0.05 (95%) was used to assess correlations between the objective and subjective ratings of the MI sessions. The software used was IBM SPSS Statistics version 27 [37].

First, the MITI rating for *cultivating change talk* was correlated with the corresponding questions answered by school nurses (question 1) and parents (question 1). Then, school nurses’ and parents’ answers to question 1 were tested for correlations. Secondly, the MITI rating for *empathy* was tested against ratings from school nurses (question 2) and parents (question 2). Then, school nurses’ and parents’ answers to question 2 were tested for correlations. Lastly, the MITI rating for *reflections-to-questions ratio* was tested for correlations in relation to the corresponding answer from school nurses (question 3).

#### 2.5.2. Qualitative Analysis

A qualitative content analysis was conducted on the interviews with an inductive and manifest approach as described by Elo and Kyngäs [38]. Transcripts were imported into NVivo 12 Plus software [39]. Initially, interviews with school nurses and parents were analysed as two separate domains. All transcripts were read-through several times, and wordings corresponding to the study aim were marked and labelled, producing codes. The codes were read-through several times and then sorted into groups, with codes belonging together forming subcategories. Secondly, subcategories were investigated and grouped into an emerging pattern of hierarchically structured categories. Finally, the two separate domains were collapsed, with related subcategories from both domains merged under mutual generic categories. The qualitative analysis was an organic process, with the authors working together, discussing, and revising formulations of categories throughout the process. All transcripts from parent interviews were read and coded by two authors (M.M. and Å.N.), and all transcripts from school nurse interviews were read and coded by two authors (M.M. and S.A.). To further ensure credibility of the analysis, two of the co-authors (Å.N. and S.A.) coded one transcript from each domain. Similarities and differences were discussed through peer debriefing. Confirmability was addressed with constant references to the raw data for quotes (audit trail) from both domains (parent and school nurse) to illustrate emerging subcategories and categories. M.M. drafted and revised the manuscript, and all qualitative co-authors (M.M., Å.N., and S.A.) read and approved the final results, text, and phrasings [36,40]. The qualitative parts of this study are reported in accordance with COREQ checklist [41], can be found as a supplementary file to this manuscript.

To enhance reflexibility and to avoid unintentional bias, it is important to provide an understanding of the researchers’ perspectives and background [42]. M.M. is a registered nurse with a background in public health. She has worked as a primary school nurse and is currently a PhD student at the department of Global Public Health at Karolinska Institutet. S.A. holds a PhD, is an experienced qualitative researcher with a background in anthropology and public health, and currently works as a lecturer in department of Nursing at Karolinska Institutet. H.L. holds a PhD, has a background in political science and psychology, and is an expert in behaviour change interventions using motivational interviewing. She is currently the head of the MI Quality Assurance coding lab at Karolinska Institutet, which, e.g., trains health care practitioners in applying MI and evaluates practitioners MI performance using the MITI protocol. Å.N. holds a PhD and is an experienced qualitative researcher with a background in anthropology, behavioural science, and public health. She is a senior lecturer in the department of Clinical Neuroscience at Karolinska Institutet and the department of Psychology at Stockholm University. Her expertise lies in behaviour change and health-promoting interventions focusing on parenting practices. Both Å.N. and H.L. are MI trainers and members of the Motivational Interviewing Network for Trainers (MINT).

#### 2.5.3. Integrated Analysis

After the qualitative and quantitative data had been analysed, as described above, the findings were merged by comparing statistical findings with qualitative findings [43]. This was accomplished through close collaboration whereby two of the authors (M.M. and Å.N.) discussed the essence of the qualitative and quantitative findings and then merged these into a joint display of findings. The proposed display was reviewed by the two other authors (S.A. and H.L.). The final version of the display was approved by all authors. The integrated results are presented in a joint display to expand understanding and to illustrate the relations of how objective and subjective ratings of the MI sessions resonated with school nurses’ and parents’ perceptions of the conversations [44].

## 3. Results

### 3.1. Participant Characteristics

Participating nurses (*n =* 7) were, on average, 47 years old and had worked for an average of 3.3 years as a school nurse. Altogether, the school nurses conducted and rated 97 MI sessions. Of the parents who participated in the sessions, 65 (47 mothers) rated the MI sessions through the distributed survey. All 7 school nurses and 17 parents (10 mothers) were interviewed about their experience of the MI sessions (Table 1).

### 3.2. Ratings and Correlations

The means of the objective and subjective ratings and correlations are presented in Table 2. Regarding the mean values, parents (*n =* 66) generally rated nurses’ ability to cultivate change and *empathy* higher than both nurses themselves (*n =* 97) and MITI coders (*n =* 89). Regarding correlations, no significant correlations between the ratings reflecting nurses’ ability to cultivate change were found. Furthermore, considering MITI ratings of *empathy*, no significant correlations were found with either nurses’ or parents’ ratings. Nonetheless, parents’ ratings of nurses’ *empathy* significantly correlated with how nurses themselves rated their *empathy* competence (*r =* 0.29, *n =* 66, *p* = <0.05). Furthermore, nurses’ ratings were found to significantly correlate with objective MITI ratings regarding the *reflections-to-questions ratio* (*r =* 0.41, *n =* 97, *p* = <0.01).

### 3.3. Qualitative Findings

Qualitative analyses generated two generic categories: ‘*meeting the other’* and ‘*perceived quality’*. In the school nurses’ domain, *meeting the other* consisted of two subcategories: ‘*shifting power relations in sensitive meetings’* and ‘*just taking the time to listen and confirm’*. Corresponding subcategories derived from parent interviews were ‘*respectful and professional’* and ‘*person-centred–or not’.* The category ‘*perceived quality’* was built up by school nurse subcategories *‘mastering MI as a method’* and *‘challenges and lessons learnt’*, as well as the subcategory *‘motivated and empowered’* from the parents’ domain (Table 3).

#### 3.3.1. Meeting the Other

School nurses and parents expressed how the health conversation as part of the HSSP provided a sanctuary for meeting the other. Although topics discussed during the meeting were sometimes perceived as sensitive, both parties welcomed the opportunity to discuss pressing issues for the individual family.

##### Shifting Power Relations in Sensitive Meetings

The school nurses described how using MI provided a more equal power balance in the conversation. For example, parents were described as more involved and as the obvious experts on their child and their families’ own circumstances, whereas the school nurses’ function was to act as a mirror rather than directing and giving advice. One school nurse described the shifting power balance:

*“There was no lecturing from me, no finger-wagging [Swe: pekpinnar] from the school nurse so to speak...the parents were involved in a different way than just me sitting and lecturing”*.

(School nurse 1)

However, asking questions about family habits could sometimes feel intrusive or culturally inappropriate. Conversations about children’s weight development were described by the school nurses as extra sensitive, and they were afraid to generate feelings of guilt in the parent. Talking about the child’s habits regarding activity, screen time, or food intake was perceived to be easier and less stigmatizing:

*“No, it’s much easier of course to talk about screen time or about eating candy every day or something like that, but the weight is very loaded... I think some parents feel guilty or they take it personally... that they are bad parents who let their child become, get obese, develop obesity”*.

(School nurse 1)

##### Respectful and Professional

Parents’ described meetings as positive when the school nurse was perceived as respectful and professional. Examples include descriptions of a school nurse that was nice and easy to get along with, or who was perceived as pedagogical and well-informed. Other factors for a positive meeting include a stress-free and tolerant atmosphere, as well as a school nurse who was adaptive to parents’ schedules. One father explained the meeting like this:

*“Yes, it went fantastically well. Yes, she was amazing, she gives information calmly and it was not stressful and on a good level. I remember this conversation, it was really-really great.”*

(Father 1)

Parents perceived the meetings as less professional when the school nurse did not feel prepared, the conversation felt stiff and forced, or when the atmosphere in the room felt strange:

*“She [the school nurse] had some papers in front of her that she followed, but it felt... I do not know, stiff and strange”.*

(Mother 1)

##### Just Taking the Time to Listen and Confirm

School nurses described how the conversation contributed to a special bond with the parents. Signs that the parents appreciated the meeting were sometimes subtle, such as a nice atmosphere or that the parents smiled after the conversation. Parents spontaneously greeted or stopped to talk in the hallway, wanting to share how everything went or to ask for further advice. School nurses described how some parents vividly expressed gratitude in the form of hugs, crying, or kind words:

*“She [the mother] kind of threw herself and hugged me really hard. So, that’s a little bit what I mean, to be listened to. I think most parents experienced that during the conversation, that they actually got the chance and the time”*.

(School nurse 2)

In some cases, school nurses perceived that parents already had knowledge and did enough with regards to children’s healthy behaviours, whereas other parents expected ready-made solutions for the child instead of figuring it out themselves in line with the MI spirit:

*“…then there was some [parents] in my opinion, who didn’t get much out of the visit. Because it was all about [the parent] figuring things out, and many peoplejustwant things served like, that you [the school nurse] should have a ready solution, like this is what you should do”*.

(School nurse 3)

##### Person-Centred—Or Not

Some parents described how the conversations were perceived to be adapted specifically for them, how they directed the conversation by talking about what was important to them, and that the school nurse supported and gave advice based on what the parent chose to talk about:

*“I thought she [the school nurse] felt very informed and yes, like not “pushy” in any way, but rather that I should come up with solutions and things like that. It wasn’t like a lecture. I had to think and reflect more myself. That’s what I thought was good... I was leading the conversation. It wasn’t like she [the school nurse] was in charge, but I kind of got to talk about what I was experiencing and if there was anything that I could change and improve on. Like that. I got some support, but that I had to think myself about what I could do to improve our situation as a family”*.

(Mother 2)

Other parents felt misunderstood or accused of having failed in their parenting. Examples were when the school nurse claimed a need for change of many behaviours or when parents wanted more concrete support from the school nurse rather than finding solutions themselves:

*“No, it was more that it [the MI session] didn’t give anything new. It was more to state that ‘yes, she eats as she does, is alert and energetic and she eats what she wants.’ Yeah, I don’t know it was like, nothing concrete. (Interviewer: “How would you have liked it?”) Well, to get better advice on how to get her to eat a little differently”*.

(Mother 3)

#### 3.3.2. Perceived Quality

School nurses reflected on their own MI performance, whereas parents described how the conversation sparked their motivation to make healthy choices for their family.

##### Mastering MI as a Method

For the school nurses, MI was a new way of thinking about routine health visits. Some school nurses struggled to change the existing routine, whereas others found it a natural progression of their profession. One school nurse described it this way: *“If you are a beginner when it comes to motivational interviewing, it will be a bit, well it will be a bit awkward”* (School nurse 3). In general, perceptions were that the initial conversations were of poorer quality and that the quality improved after supervision and with more experience in using the method. Some nurses perceived how that the quality of the conversations could vary from time to time:

*“Sometimes it felt great, and I experienced a good flow in the conversation, and sometimes it just felt like this just went pear-shaped [Swe: ‘skit och pannkaka’], there was no MI whatsoever. And that’s probably perfectly normal, but still…”*

(School nurse 4)

Some parts of MI were perceived as more difficult to implement than others. In general, affirmations and simple *reflections* were perceived as easy to deliver, whereas complex reflections and promoting change talk were perceived more difficult:

*“I had a hard time finding this change talk, and I ended up in a more supportive role. So, I really had to work to remember, have mine, have a small paper with supporting notes in front of me and things like that... Not to miss the change talk”*.

(School nurse 5)

##### Motivated and Empowered

Most parents appreciated the conversation with the school nurse. Whereas some parents found that the meeting gave them nothing new, other parents described how the school nurse gave concrete advice on strategies that became a catalyst for improved behaviours:

*“It [the MI session] was like an eye-opener, even at the first meeting. You always had it somewhere subconscious, but it was only after this conversation with the school nurse, all these questions and these ideas about how to improve and what you could do. That’s when I got this commitment, and the motivation to deal with this [healthy behaviour change], so to speak.”*

(Father 2)

Parents described a sense of reassurance when the school nurse felt available for follow-up questions or confirmed that the child was monitored after the conversation. Some parents described how they felt empowered in their parenting when the school nurse confirmed that they had good knowledge or habits regarding the child’s food and activity behaviours:

*“Well...you got confirmation that you were on the right path, and you got to know things like... Even though you’re a parent, you don’t know everything and sometimes it’s nice to just be able to listen to others perspective. So, it was like a reassurance from her [the school nurse]”*.

(Mother 4)

##### Challenges and Lessons Learnt

School nurses described success factors for high-quality MI sessions. Examples include scheduling enough time, creating a calm atmosphere in the room, and planning so that the child or younger siblings did not have to stay in the room during the conversation. On the other hand, several school nurses described how it was difficult to use MI when the parent did not understand Swedish well enough or needed to use an interpreter. In these cases, MI quality was difficult to assure because of how the conversations were understood or what was translated:

*“This MI was very difficult [with interpreter], I couldn’t do it. I know I tried at some point, but I then understood that the interpreter had, from the reaction of the parents, that they had got it wrong, so that I kind of had to give that [using MI with interpreter] up a little bit”*.

(School nurse 1)

*“When you need to have these nuances in the conversation. Like, how does the interpreter affirm, how does the interpreter translate my reflections and affirmations, you know…”*.

(School nurse 6)

Experiences were scattered regarding MI sessions with parents who had no explicit problem area. Some school nurses described how they successfully managed to empower these parents by confirming the established healthy behaviours, whereas other school nurses expressed a frustration over the conversations not proceeding or not finding a problematic behaviour to focus on:

*“There was one mother that really moved me. Because she thought she was a terrible mother, but she did so much, and she had tried so hard. And for me [the school nurse] just to be able to confirm and see her. She [the mother] was sitting here crying at the end, because she felt ‘No, I’m not such a bad mom after all’”*.

(School nurse 6)

### 3.4. Joint Display of Findings

Three overarching joint concepts emerged when combining the results in a joint display: recognise and cultivate parents’ motivation, ability to listen and reflect what parents say, and show consideration for parents’ worldview (Table 4).

#### 3.4.1. Recognise and Cultivate Parents’ Motivation

The joint concept *recognise and cultivate parents’ motivation* comprised the quantitative ratings regarding school nurses’ ability to *cultivate change talk* ang the qualitative generic category *perceived quality.* Findings revealed that although MITI ratings were low and school nurses were somewhat critical of their MI performance, parents had left the meeting feeling motivated and empowered to promote their children’s healthy behaviours. The same viewpoint was expressed by the school nurses, who perceived that the MI sessions had been useful when motivating parents. Nonetheless, none of the ratings regarding nurses’ ability to *cultivate change* correlated. Objective MITI scores were rated the lowest, and nurses’ ratings were slightly higher. Parents, on the other hand, generally rated the conversations higher than both MITI and school nurses with regards to nurses. School nurses’ ambivalence in rating their own performance particularly resonated with the qualitative findings, with descriptions of challenges learning the method and how the perceived MI performance shifted depending on parents’ tangible interest. Furthermore, qualitative findings reflected the same sense; several parents described how they had left the meeting feeling motivated and empowered to commit to change less healthy behaviours or sustain already healthy behaviours in their children.

#### 3.4.2. Ability to Listen and Reflect what Parents Say

Findings regarding the joint concept of *ability to listen and reflect what parents say* suggest that the MI skill *reflections* were easier for school nurses to deliver during the conversations and to self-rate. This was evident, as school nurses’ ability to estimate their own *reflections-to-questions ratio* correlated significantly with the objective MITI ratings. Qualitative findings suggest that the school nurses had started to reflect and critically evaluate their own performance, resonating with the quantitative findings. Moreover, the school nurses expressed how they perceived some MI-specific technical skills, such as *simple reflections* and *affirmations*, easier to recognise and deliver in the conversations, whereas struggles were expressed regarding other MI techniques, such as *complex reflections* and *cultivating change talk*.

#### 3.4.3. Show Consideration for Parents’ Worldview

The joint concept of show consideration for parents’ worldview included the objective and subjective ratings of school nurses’ empathy, together with qualitative findings regarding parents’ and school nurses’ expressions of meeting the other. Both school nurses and parents perceived the meeting as respectful and family-centred and, as such, helpful in building a trusting relationship. Perceived competence in empathy as scored by school nurses and parents correlated in the statistical analysis. These similarities were also observed in the qualitative results and reflected in multiple subcategories. For example, both school nurses and parents described how the meeting seemed appreciated, how parents’ agendas were in focus, and how a more balanced power relation contributed to a respectful and professional meeting.

## 4. Discussion

With this study, we aimed to explore school nurses’ MI competence using quantitative, objective, and subjective ratings assessed by MITI coders, parents, and school nurses, together with qualitative perceptions of delivering and participating in MI session as reported by school nurses and parents. Altogether, our quantitative and qualitative findings show that school nurses’ MI performances were rated and perceived as valuable and family-centred by both school nurses and parents, who had left the meeting feeling motivated and empowered to promote their children’s healthy behaviours. Nonetheless, school nurses were critical of their own MI technical performance, and they found that reflections were easier to deliver and to self-rate.

### 4.1. Recognise and Cultivate Motivation

When it comes to subjective ratings of MI skills, previous research indicates that practitioners’ self-reported skills often are more positive than objective rates regarding MI adherence and competence and that self-rated skills might not predict the ability to practice MI [45]. This was also true for school nurses in our study, who rated their performance regarding *cultivate change* and *empathy* higher than MITI. The joint display of integrated statistical outcomes, together with qualitative findings, might elucidate why school nurses and parents rated the MI conversations higher than the objective MITI coders regarding *cultivate change* and *empathy.* Qualitative findings suggest that although MITI ratings, in general, were low, both parents and school nurses perceived the conversations as respectful and family-centred. This knowledge is, in turn, valuable in terms of informing how MI educators could tailor the curriculum to suit the practitioners’ perceived and objectively observed difficulties in *cultivating change talk*.

Furthermore, our findings suggest that both school nurses and parents perceived MI as valuable for motivating parents to promote healthy behaviours in their child. These findings reflect school nurses’ awareness and insight into what constitutes core MI skills and that they had started to reflect upon their own performance after MI training. Parents, on the other hand, perceived the school nurse as skilled and professional. This was evident in both parents’ quantitative ratings and qualitative descriptions of how they had left the meeting *feeling motivated and empowered* as parents. These findings are similar to those reported in previous research that has shown that although school nurses did not reach established cutoffs for MI proficiency, *cultivate change talk* and *reflections-to-questions ratio* were associated with decreased intake of unhealthy foods in children when MI was performed together with parents [33]. In sum, evidence is growing to support the suggested cut-offs for MI proficiency [23] might not be suitable for all contexts and that in the health-promoting context, lower MI skills could also be beneficial and appreciated. Promising behaviour change and sustainment of healthy behaviours, as well as clients’ positive perceptions, have been observed at lower MI proficiency levels than suggested when MI was used in a health-promoting context. Nonetheless, more research is needed for correct assumptions to be drawn.

### 4.2. Ability to Listen and Reflect

Results from our study suggest that although school nurses’ ratings of *cultivate change* and *empathy* did not correspond to objective ratings, the self-rated *reflection-to-question ratio* rendered more accurate estimation ratings when compared to objective MITI ratings. Previous research revealed that MI sessions with more reflections than questions (*reflection-to-question ratio*), is associated with higher scores of clinician *empathy* [46]. One way to better understanding the mechanisms of MI could be to further associate MITI scores, together with practitioners’ and clients’ ratings to clients’ actual behaviour change, using validated and reliable scales developed for this purpose [21,47]. Furthermore, research exploring clients’ perspectives and experiences of participating in MI sessions is lacking.

### 4.3. Show Consideration of Worldview

Parents’ and school nurses’ ratings in this study were correlated with regard to nurses’ relational performances. These findings correspond to a previous study wherein physicians’ self-rated *empathy* was association with patient satisfaction in emergency wards, where a positive correlation was found [48]. This was also evident in the qualitative findings; wherein relational competence was found to be an appreciated aspect in the conversations. In relation to the present study, the differences seen regarding perceived MI quality between objective and subjective ratings, together with parents’ perceived quality of the MI sessions, could be interpreted as one explanation for the previously observed behaviour change with lower levels of MI competence [23].

### 4.4. Implications and Future Research

Future research should focus on relating participants’ subjective ratings of MI with clients’ behavioural outcomes, as client experiences of participating in MI sessions is considerably understudied. Moreover, some school nurses expressed how it was challenging to meet families with no clear problem behaviour. Future MI training designed for practitioners active in settings with a general population should emphasise MI skills aimed at sustaining clients’ healthy behaviours rather than only focusing on eliciting behavioural change.

### 4.5. Strengths and Limitations

This study includes a broad variety of data, both qualitative and quantitative, that provide a broad picture of how school nurses, parents, and objective MI assessors perceive the same conversations. Furthermore, the mixed-methods design further extends the understanding of the different viewpoints with regard to the perceptions of delivering and participating in MI.

One limitation could arguably be the limited and homogenous study sample of interviews due to the convenience sampling strategy of parents, as well as the relatively thin data collected through one-on-one interviews rather than focus groups. This strategy considered only school nurses’ perceptions regarding MI conducted through an interpreter, and parent perspectives should be researched further.

In addition, the validated scale for measuring client perceptions of motivational interviewing (CPMI) developed by Madson and colleagues [49] was, unfortunately, not available when data collection was undertaken for this study. Therefore, the questions used were developed to reflect parents view on *cultivate change talk* and *empathy*. In retrospect, the question used for measuring parents’ views on cultivating change did not correspond to the nurses’ questions and might have influenced how the ratings correlated. Additionally, more ratings comparable to the MITI protocol could have been added to the subjective ratings.

## 5. Conclusions

School nurses’ MI performances were rated and perceived as valuable and family-centred by both school nurses and parents, who had left the meeting feeling motivated and empowered to promote their children’s healthy behaviours. Nonetheless, school nurses were critical of their own MI technical performance, and they found that reflections were easier to deliver and to self-rate. Overall, MITI ratings were the lowest, and those of parents were the highest.

## Figures and Tables

**Table 1:** Descriptive characteristics of participating school nurses (*n =* 7) and parents (*n =* 100).

	Attended MI Session	Rated MI Sessions	Interviewed
	*n* (%)	*n* (%)	*n* (%)
School Nurses	7	97	7
Women	7	7	7
Mean age	47	47	47
Previous MI education (yes)	3 (43)	3 (43)	3 (43)
Years active as school nurse	3.3	3.3	3.3
Parents	99	65	17
Mothers	65 (65)	47 (72)	10 (59)
Education level (low) ^1^	27 (27)	17 (26)	4 (23)
Born outside the Nordic region ^2^	65 (65)	42 (65)	11 (65)
Children (of participating parents)	97	65	17
Girls	48 (48)	31 (48)	9 (53)
Mean age	6.3	6.3	6.3
Weight status (overweight or obesity) ^3^	25 (26)	18 (28)	5 (29)

^1^ Education level: defined as <12 years of reported formal education; ^2^ Nordic region: Sweden, Norway, Denmark, and Finland; ^3^ weight status: height and weight adjusted for age; cut-offs based on BMI-SDS.

**Table 2:** Mean objective and subjective ratings of school nurses’ MI skills with standard deviations, range, and correlations.

Variable and Respondent	*n*	M (SD)	Range	1	2	3
Cultivate Change						
MITI (1)	89	1.7 (0.8)	1–4	1	0.06	0.13
School nurse (2)	97	3.0 (0.8)	1–5	0.06	1	−0.20
Parent (3)	66	4.6 (0.7)	2–5	0.11	−0.20	1
Empathy						
MITI (1)	89	2.1 (0.9)	1–4	1	0.91	0.81
School nurse (2)	97	3.5 (0.8)	1–5	0.91	1	0.29 *
Parent (3)	66	4.7 (0.5)	3–5	0.81	0.29 *	1
Reflections vs. Questions						
MITI (1)	97	0.9 (0.5)	0–2.4	1	0.41 **	-
Nurse (2)	97	1.8 (0.7)	1–3	0.41 **	1	-

* *p* < 0.05 (two-tailed), ** *p* < 0.01 (two-tailed).

**Table 3:** Qualitative results, presented by generic categories with subcategories separated by two domains: school nurses and parents.

**Categories**	**Sub-Categories**	**Domains**	
		**School Nurses**	**Parents**
**Meeting the other**		Shifting power relations in a sensitive meeting	Respectful and professional
		Just taking the time to listen and confirm	Person-centred—or not
**Perceived quality**		Mastering MI as a method	Motivated and empowered
		Challenges and lessons learnt	

**Table 4:** Joint display of quantitative and qualitative findings, presented as joint concepts with respective correlations, and linked generic categories and sub-categories with illustrative quotes.

Joint Concepts	Correlations				School Nurses’ Perceptions	Parents’ Perceptions
	MITI vs. SN (*r*)	MITI vs. Parent (*r*)	SN vs. Parent (*r*)		Generic Category	
					Perceived Quality	
**Recognise and cultivate parents’ motivation** Quantitative variables: - Cultivate change talk Qualitative category: - Perceived quality	0.17	0.13	−0.10	Sub-categories and quotes	**Mastering MI as a method** *”I had a hard time finding this change talk, and I ended up in a more supportive role”* (**School nurse 5**) **Challenges and lessons learnt** *“MI was very difficult [with interpreter], I couldn’t do it”* (**School nurse 1**)	**Motivated and empowered** *“It was only after this conversation with the school nurse… I got this commitment, and the motivation to deal with this [healthy behaviour change]”* (**Father 2**)
**Ability to listen and reflect what parents say** Quantitative variables: - Reflections Qualitative category: - Perceived quality	0.40 **	n/a	n/a		**Mastering MI as a method** *“Simple reflections are one thing, but when you need to use, what do you call them, advanced reflections, those are somehow more difficult”* (**School nurse 6**)	n/a
**Show consideration for parents’ worldview** Quantitative variables: - Empathy Qualitative category: - Meeting the other	0.03	0.14	0.25 *		**Shifting power relations in a sensitive meeting** *“There was no lecturing from me, no finger-wagging from the school nurse so to speak”* (**School nurse 1**) **Just taking the time to listen and confirm** *“So, that’s a little bit what I mean, to be listened to… they [the parents] actually got the chance and the time”* (**School nurse 2**)	**Respectful and professional** *“Yes, she [the school nurse] was amazing, she gives information calmly and it was not stressful and on a good level”* (**Father 1**) **Person-centred—or not** *“It wasn’t like she [the school nurse] was in charge, but I kind of got to talk about what I was experiencing”* (**Mother 2**)

* Indicates *p* < 0.05 (2-tailed) ** indicates *p* <0.01 (2-tailed).

## Data Availability

Data are not available for download in order to protect the confidentiality of the participants. The data are held at Karolinska Institutet.

## Supplementary Materials

The following supporting information can be downloaded at: https://www.mdpi.com/article/10.3390/clinpract12030039/s1: COREQ (COnsolidated criteria for REporting Qualitative research) Checklist.
